# Smart parking systems: comprehensive review based on various aspects

**DOI:** 10.1016/j.heliyon.2021.e07050

**Published:** 2021-05-15

**Authors:** Abrar Fahim, Mehedi Hasan, Muhtasim Alam Chowdhury

**Affiliations:** a Department of Electrical and Computer Engineering, North South University, Dhaka 1229, Bangladesh; b Department of Electrical and Electronic Engineering, University of Science and Technology Chittagong, Chattogram 4202, Bangladesh

**Keywords:** Smart parking system, Internet of things, Wireless sensor network, VANET, Sensor, Image processing, Smartphone application, Web application, Machine learning, Neural networks

## Abstract

Parking allocation has become a major problem in modern cities for which numerous smart parking systems (SPS) have been developed. This paper aims to provide comprehensive study, comparison and extensive analysis of SPSs in terms of technological approach, sensors utilized, networking technologies, user interface, computational approaches, and service provided. Moreover, the paper fills up the research gap by providing a clear insight into the suitability of SPSs in various environmental conditions and highlights their advantages/disadvantages. The extensive comparison among multiple aspects of SPSs would enable researchers, designers, and policymakers to identify the best suited SPS and understand the current trends in this sector.

## 1 Introduction

The modern world is changing rapidly which is fueled by scientific discoveries and technological inventions that facilitate the creation of numerous smart devices, appliances, and systems. These intelligent devices, appliances, and systems include home apparatus, robots, intelligent vehicles, smart transportation systems, automation, smart sensor networks, communication systems, and various other gadgets. Through these technological blessings, human life has become more accessible, flexible, and comfortable. Nowadays, various human life aspects are either entirely or partially affected by modern technology and its blessings.

Over the last few years, Internet of Things (IoT) has utterly altered habitual human behavior by providing them with numerous facilities and comfort options to have ease in everyday life. Equipped with an Internet connection and sensor networks, electronic devices in the digital world are connected through IoT technology [1, 2]. Research conducted on the prospects of IoT has figured out an exponential increment of IoT devices. As per estimation, more than 25 billion IoT-based appliances will be connected to the internet by the end of 2020 [1]. IoT enables integration, interaction, and communication with digital electronic devices, sensors, and actuators which provide the required services to attain specific goals more efficiently [3, 4]. IoT's security uses various security measures, making it a matrix for other technological advancements [5].

Due to the exponential growth and development of IoT and cloud-based smart systems, the concept of developing smart cities has gained a new dimension. The goal of Smart City is to reduce operational costs, improve city management, enhance effectiveness, and improve productivity [6]. The concept of a smart city includes systematic monitoring and management of infrastructure, building, intelligent transportation system [7], healthcare [8, 9], education, energy consumption, public security [10].

SPS is an essential element of the transportation system in the smart city concept. In highly compact and densely populated sectors in urban areas, scarcity of parking space is a major problem. According to the authors in [11], around 30% of the vehicles on the roads of major cities are manually searching for vacant parking lots and it takes around 7.8 min to find a suitable parking lot. The statistical figure mentioned above tells about the severe wastage of time and the initiation of traffic congestions around the big cities. It also causes fuel wastage, driver frustration, and air pollution [12]. Corresponding to [13, 14], traffic congestion affects the fuel consumption rate. As a result, the emission of Carbon Monoxide (CO), Carbon Dioxide (CO_2_), Volatile Organic Compounds (VOCs), Hydrocarbons (HCs), and Nitrogen Oxides (NO_x_) increases, which result in air pollution. As stated by the United Nations Environment Program (UNEP), around 7 million premature deaths were related to air pollution globally [15]. Another study led by researchers of the Harvard School of Public Health estimates that traffic congestion will have an economic impact of $100 billion by 2020 [16].

An estimated $13 billion will be spent in 2020 as health costs due to traffic congestion in the USA and this number is estimated to become $17 billion by 2030 [16]. On the other hand, according to the Australian infrastructure audit 2019 [17], the total cost due to road congestion in the year 2016 in Australia was around $19 billion, which is estimated to reach $39 billion by the year 2031. Smart parking systems can be a sound solution to the reduction of traffic congestions, which, in turn, will reduce air pollution and the health risks associated with air pollution.

The rapid advancement in the internet, communication, and information technology have paved the way for developing efficient smart parking systems at a relatively lower price. Due to this reason, many researchers have implemented various SPS based on different approaches and sensors. Considering the facts mentioned above, the authors in [18] reviewed various smart parking solutions developed by researchers by providing short descriptions about their advanced solutions. The paper tabulates the advantages and disadvantages of the multiple solutions alongside with the sensors and networking tools required to develop various SPSs. But, the paper describes very little about the methods or approaches which pave the systems to provide the services to the user. Similarly, Nene et al. described vehicular parking systems based on recent smart parking technologies [19]. The paper provided a brief description of the SPSs and compared them to highlight their strengths and limitations.

A review on open parking lot suitability in SPSs has been provided in [20]. Here, the authors talked about various smart parking tools, sensors and approaches to develop SPSs. The paper provided a list of suitable sensors for only open parking lots and talked about their strengths and drawbacks. Therefore, depending on functionality, certain SPSs are effective in certain conditions. As a result, it becomes necessary to compare SPSs based on methods, sensors, networking techniques, computational approaches, and services. As described earlier, the review articles lacked some of the aforementioned core parts or were somewhat unsuccessful in portraying the overall SPS concept in a bird's-eye view to both technical and non-technical people. However, this research utilizes the SPS literature surveys to fulfill all the missing parts of the review papers mentioned above by illustrating:

• the various methods or approaches used by different SPSs developers and their suitability towards different parking conditions
• the sensing tools used by SPSs alongside with their pros and cons
• exhaustive review and classification of SPS according to various aspects such as networking technologies, user interfaces, computational approaches, and provided services.
• a comprehensive analysis by providing advantages and disadvantages of existing SPSs.

Hence, this research aims to aid researchers, system designers, and policymakers by providing a comprehensive and systematic comparison among various SPSs. Moreover, the advantages and disadvantages of the SPSs have been extensively analyzed and highlighted to enable anyone to understand and choose the most appropriate SPS. Besides, an extensive review of SPSs will allow new researchers in the relevant field to get an overall idea of the current research trends.

**Figure 1:**
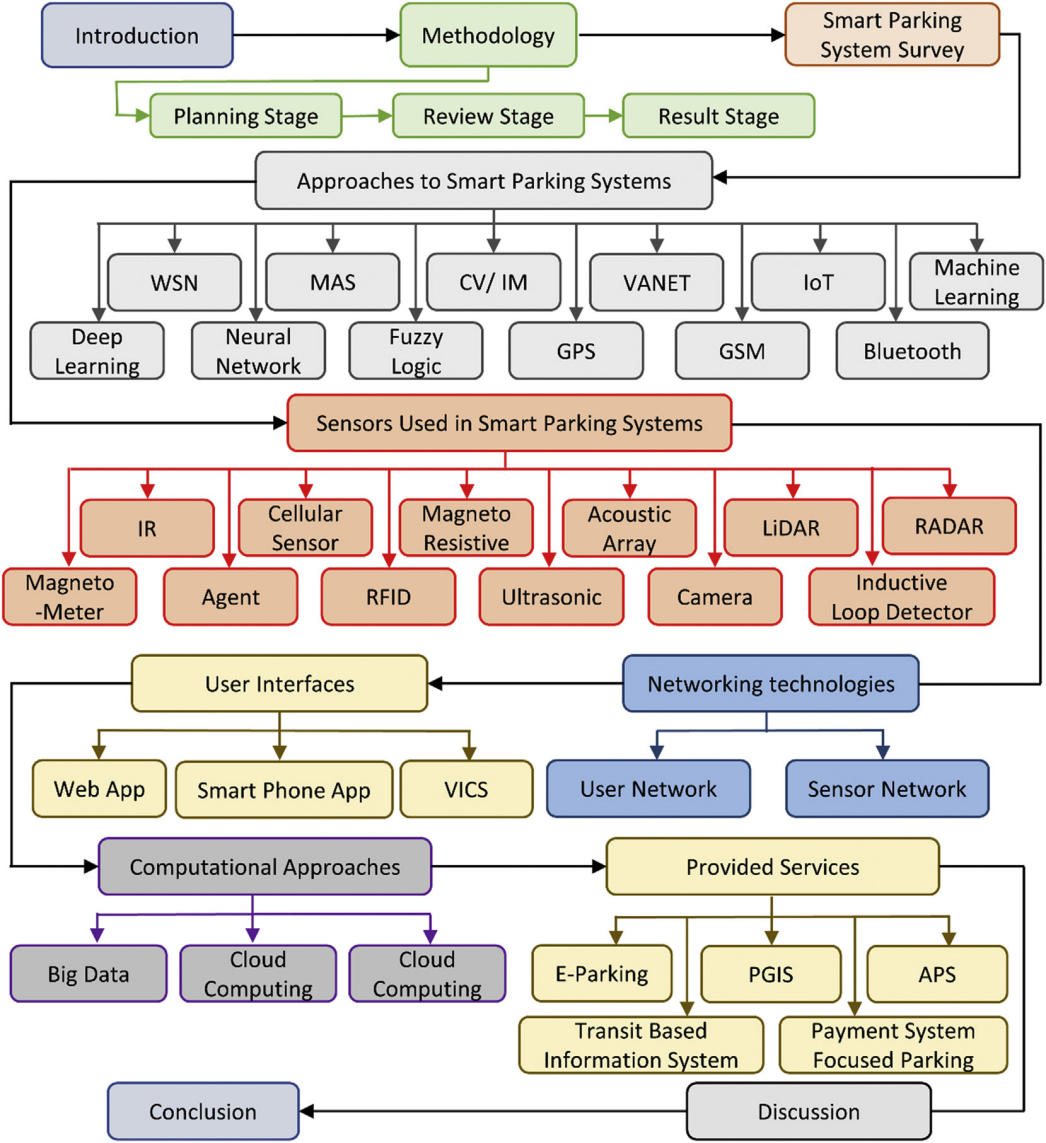
Organization of the paper.

Figure 1 illustrates the overall organization of the paper as a block diagram. The paper is structured in the following manner. Section 1 introduces the paper and presents the motivations for performing extensive study in SPS. Section 2 provides a discussion on the methodology taken in this paper to review the existing literature. The methodology to review existing literature is divided into three stages: planning, review and result. An extensive literature review of existing SPSs has been provided in Section 3. Based on the approaches, section 4 classifies the SPSs into 12 different groups. These groups are Wireless Sensos Networks (WSN), Multi Agent Systems (MAS), Computer Vision (CV)/Image Processing (IM), Vehicular Adhoc Newwork (VANET), Internet of Things (IoT), Machine Learning, Deep Learning, Neural Network, Fuzy Logic, Global Positioning System (GPS), Global System for Mobile (GSM) and Bluetooth based SPS. In Section 5, a review of sensors used in SPSs has been provided. According to sensors, the SPSs have been classified into 12 groups. Based on sensors, SPSs can be classified into Infrared Ray (IR), Cellular Sensor, Magneto-Resistive, Acoustic Array, Light Detection and Ranging (LiDAR), Radio Detection and Ranging (RADAR), Magneto-Meter, Agent, Radio Frequency Identification (RFID), Ultrasonic, Camera and Inductor Loop sensor based systems. The networking part of the smart parking systems has been examined in Section 6. Based on networking technologies, SPSs can be classified into two groups namely user network and sensor network. Section 7 talks about the communication interfaces between the system and the user. The user interfaces can be classified as web application, smart phone application and vehicle information and communication (VICS). Different computational approaches taken for developing various SPSs are mentioned in section 8. The main computation approaches are Big Data, Cloud Computing and Fog Computing. Section 9 categorized SPSs based on the services they provide. The main services that SPSs consist are electronic-parking (E-Parking) system, parking guidance and information system (PGIS), automated parking system (APS), transit based information system and payment focused parking. In section 10, a comprehensive analysis and discussion of the reviewed SPSs is given based on the factors provided in previous sections. Finally, concluding remarks have been stated in section 11.

## 2 Methodology

This section narrates the methodologies adopted in this research work to review the existing works and create a bird's eye view of the SPS concept. To gather existing information on SPS, this research has considered reputed publishers such as IEEE Xplore, ScienceDirect, Springer Link, MDPI, ACM Digital Library, Hindawi. This paper adopted the research method described in [21]. The adopted method categorized the paper's reviewing scheme into three major stages. The stages are planning stage, review stage, and result Stage.

**Figure 2:**
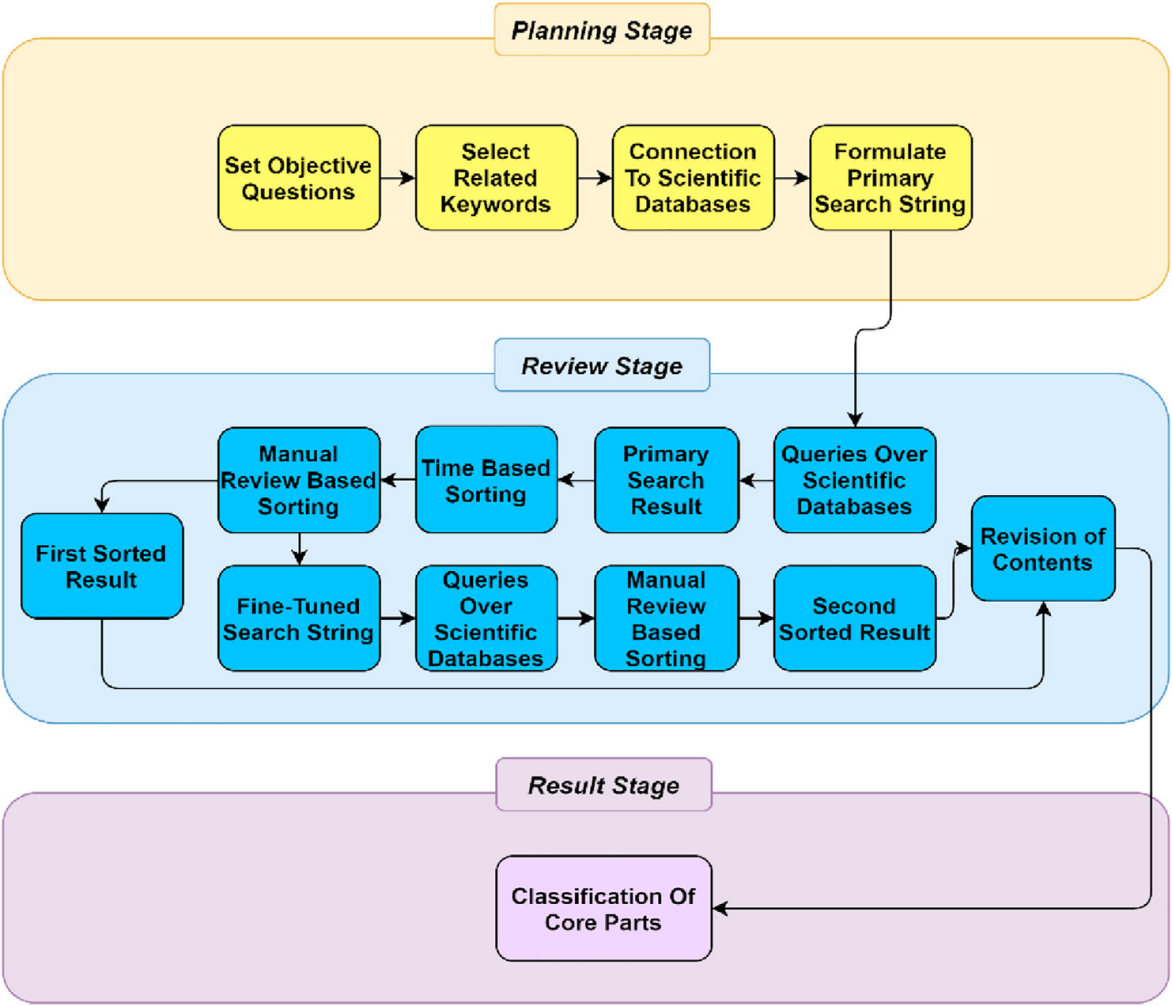
Research method in detail.

The planning stage defines guidelines to search for different review materials. The review stage focuses on strict guidelines for developing search strings to find the correct review materials from different repositories. This stage also collects preliminary results, extracts pertinent research papers, and sorts the aspirant papers. Finally, in the result stage, a thorough review of the selected documents is conducted. The overall methodological process of the literature search is illustrated in Figure 2.

### 2.1 Planning stage

The planning stage is the initial stage of the research. The stage initiates with the formulation of the questions, which pinpoints the main objective of the research. To formulate the objective-based question sets, a set of keywords needs to be selected to articulate search strings to find specific literature stored in scientific databases.

The following questions were at first established to locate the objective of this research

• What are the approaches or methods for building a smart parking system?
• What sort of sensors were used in developing a smart parking solution?
• What type of networking tools were implemented in developing a smart parking system?

The questions are connected to the main objective of this paper, which is to review the existing smart parking solutions to find the adopted approaches, sensors, and network technologies used to develop the SPSs. The questions were also used to identify keywords such as; "smart,” "parking", "system", "solution", "sensors", "networks", "methods". After that, primary search strings are developed to identify the initial literature findings from the previously mentioned online scientific databases and web searches.

### 2.2 Review stage

In the planning stage, numerous pieces of literature came out as findings from which only the papers published within the last twenty years were selected. Although in this selection, papers published within the twenty years were chosen, but the main focus was given to the papers published within the last ten years period.

After the first sorting, the chosen papers from different online repositories were manually sorted by reviewing the title, abstract, and conclusion. In this stage of sorting, a paper is selected if it included the keywords and could provide the details that might answer the set of questions regarding the objective of the paper. A paper is disregarded if it fails to portray any topic related to the aim of the paper.

In the third stage, the primary search strings, which were based on the keywords, were modified by adding some additional keywords such as: "intelligent sensors", "multiagent system", "cloud", "wireless", "autonomous", "IoT". After that, another search was made through the scientific databases using the fine-tuned search strings. This search's findings were again sent through the manual review process of the title, abstract, and conclusion. Later, all the selected contents were thoroughly revised for the result stage. The overall process of search string formulation to querying scientific databases and their search results are illustrated in Table 1.

**Table 1:** Query results from different formulated strings over various scientific databases.

Scientific Database	Primary Search String	Primary Search String Result	Modified Search String	Modified Search String Result
IEEE Xplore	((((smart parking)AND systems)AND methods)AND networks)	95	(((smart parking) OR autonomous parking) AND multiagent system)	15
			(((smart parking) OR autonomous parking) AND intelligent sensors)	279
	((((smart parking) AND systems)AND methods)AND networks)	20	(((smart parking) OR autonomous parking) AND IoT)	275
			(((smart parking) OR autonomous parking) AND wireless sensor network)	153
ACM Digital Library	"Smart" AND "Parking" AND "System" AND "Sensors" AND "Method"	65	"smart parking" OR "autonomous parking" AND "multiagent system" AND "wireless sensor network" AND "IoT" AND "intelligent sensors."	161
	"Smart" AND "Parking" AND "Solutions" AND "Sensors" AND "Method"	56		
ScienceDirect	Smart Parking System	105	"smart parking" AND (IoT, OR multiagent OR system, OR wireless OR sensor OR network, OR intelligent OR sensors)	331
	Smart Parking Solutions	49	"autonomous parking" AND (IoT, OR multiagent OR system, OR wireless OR sensor OR network, OR intelligent OR sensors)	58
Springer Link	Smart Parking System	12	"smart parking" AND (IoT, OR multiagent OR system, OR wireless OR sensor OR network, OR intelligent OR sensors)	836
	Smart Parking Solution	1	"autonomous parking" AND (IoT, OR multiagent OR system, OR wireless OR sensor OR network, OR intelligent OR sensors)	141
MDPI	smart parking AND systems AND methods AND networks AND Sensors	156	smart parking AND multiagent system AND wireless sensor network AND IoT AND intelligent sensors	0
	smart parking AND solutions AND methods AND networks AND Sensors	119	autonomous parking AND multiagent system AND wireless sensor network AND IoT AND intelligent sensors	0
Hindawi	Smart Parking System	350	smart parking AND multiagent system AND wireless sensor network AND IoT AND intelligent sensors	0
	Smart Parking Solution	66	autonomous parking AND multiagent system AND wireless sensor network AND IoT AND intelligent sensors	0

Although not mentioned in the above table, similar search strings were formulated for searching in web for more literature on SPS. For searching in web, a few more keywords such as "traffic", "congestion", "pollution" were included in the existing keywords, which increased the number of findings. In case of search in web, literature from various Scopus indexed journals and conferences are considered. The search also provided some research articles published by various government organizations.

### 2.3 Result stage

In this final stage of the paper search, all the individual results and findings related to the research were integrated for documentation. Then, those findings were used to build the core parts of this paper and determine the technological trends in smart parking systems.

**Figure 3:**
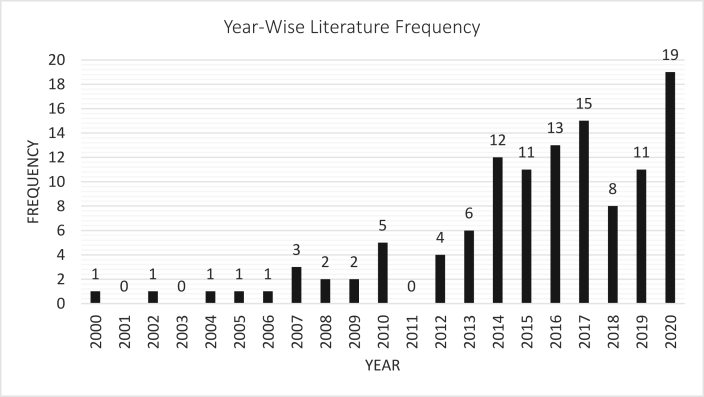
Year-wise literature publication.

**Figure 4:**
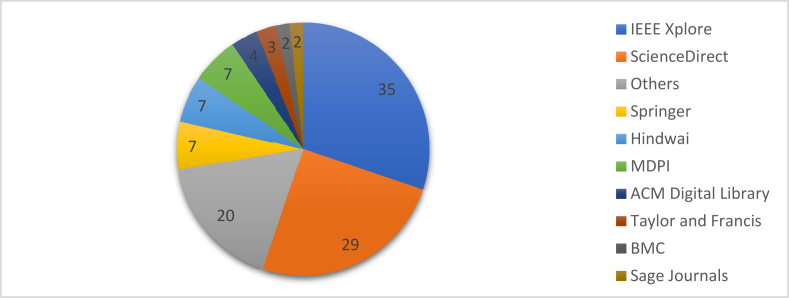
Publisher-wise literature percentage of SPS.

After the methodological process of sorting, a total of 116 pieces of literature were selected. Based on the articles reviewed in this research, two graphical representations can be illustrated. Figure 3 represents the year-wise number of literature published, and Figure 4 depicts the percentage of literature adopted from different publishers. As per Figure 3, the chosen articles were published over the span of the last twenty years. As per publisher, 4 papers were selected from ACM Digital Library, 35 from IEEE Xplore, 7 papers from MDPI, 29 pieces of literature from ScienceDirect, 7 from Hindawi, 7 papers from Springer Link, 3 works of literature from Taylor and Francis. Both BMC and Sage Journals provided 2 papers each, and the rest were taken from other publishing agencies (Figure 4). Only the articles from Scopus and Web of Science indexed journals were considered for the other publishing agencies.

## 3 Smart parking system survey

Many large cities face the dire problem of providing available parking spaces to their citizens at the peak hours of a day. As a result, citizens spend a massive amount of time searching for the perfect parking space or waiting in line to get one. This, in turn, creates traffic congestions. Considering the problems, many researchers have suggested different SPS approaches and technologies to mitigate this problem.

The authors in [22] suggested the adaptation of a cloud-based platform as a Service (PaaS) to develop an Internet of Things (IoT) based SPS. PaaS can be divided into two parts: a back-end dashboard platform and a front-end data platform. The back-end data platform provides data storage, management, and processing facility. On the other hand, the front-end dashboard platform deals with reporting and visualization of data.

In [23], a Multi-Agent System (MAS) based SPS has been designed. The system uses agent networks that coordinate between the driver and the SPS. It utilizes a negotiation algorithm that provides a negotiable parking fee depending upon different criteria. Moreover, the system offers vehicle guidance for the shortest path to the parking space and parking reservation facility.

A Car Parking Framework (CPF) based on IoT has been proposed in [24]. The framework combines an automatic car parking management system with the integration of networked sensors and actuators along with Radio Frequency Identification (RFID). The CPF provides vehicle guidance, payment facilities, parking lot retrieval, and security. The system uses a hybrid communication method instead of regular nodal communication. Due to that, the system has a low energy consumption rate with low implementation costs.

The authors of [25] developed a WSN based SPS. The system utilizes a hybrid self-organization algorithm for WSN technology. The system has been designed to be more energy efficient during wireless communication. As a result, it enhances the life expectancy of WSN nodes and overall WSN. This sort of SPS aids the users by guiding them to the nearest parking lot and the facility to reserve it. The system utilizes both web and smartphone applications to provide SPS facilities to the users.

Using Global System for Mobile (GSM), Rahayu and Mustapa proposed a secured parking reservation system [26]. The system is comprised of a security reservation module and a parking space monitoring module. The security reservation module handles the reservation of specific parking lot. The user needs to send a Short Message Service (SMS) with provides specific instructions to reserve the parking lot. The parking lot monitoring module provides the user with a layout animation of parking space occupancy status, enabling the user to choose a parking lot for reservation. The system generates a password, which is required during enter and exit points.

The authors in [27] suggested a prediction scheme of parking lot availability based on sample parking spaces. The prediction scheme uses Fuzzy logic to predict available parking spaces. The system is dedicated to the park-and-ride (PnR) commuters, which infers that the sample parking spaces are located near public transportation facilities.

Research conducted in [28] proposed a visual vehicle parking space occupancy detection method via deep Convolutional Neural Network (CNN). The system uses a decentralized approach and can detect parking lot occupancy in real-time via smart cameras. The solution datasets are compared with visual datasets.

A vision-based vacant parking space detection method has been developed in [29]. The system provides an outdoor parking service, which can detect real-time parking lot vacancies and send the parking lot's location to the driver for vehicle guidance. The system also implements the Adaptive Background Subtraction algorithm as a solution to shadow effects and changes in light.

Authors in [30] focused on a CNN based real-time parking space occupancy detection system. The CNN classifier runs on a smart camera with a very marginal amount of resources. The system is highly robust against the change of lighting conditions, effects of shadow, and partial occlusions. Results obtained from the system are compared with existing SPS for parking lot detection.

Vacant parking space detection and tracking system based on sensor fusion have been proposed in [31]. The system uses a sensor fusion technique to fuse the data produced by the Around View Monitor (AVM) system's sensors. The data generated by ultrasonic sensors are used to monitor parking space occupancy. The system has three stages: detection of parking lot markings, classification of parking lot occupancy, tracking of parking lot markings.

In [32], an SPS based on WSN and an Ultrasonic sensor has been proposed. The system has utilized the shortest path algorithm to provide the user with the shortest route towards the vacant space. Moreover, parking lot occupancy information is provided to the users.

A cloud-based SPS was proposed in [33]. The system used WSN approach to monitor parking lots of a parking area. The parking lot status is then wirelessly transmitted to the cloud server. The user can see real-time parking lot status via an android application on a smartphone.

The authors in [34] proposed an IoT-based SPS. The SPS can monitor and indicate parking lot availability to the user via IoT applications. The system has three parts: On-field Network, Cloud platform, User side platform. The on-filed network consists of vehicle detection sensors, which detect parking lot occupancy. Then, the data from the sensors are transferred to the User side platform via a cloud platform. From the user side platform, any user can easily see the parking lot status of the particular parking area.

An SPS framework based on IoT for urban areas was developed in [35], which focuses on parking reservations as the main criteria. This SPS provides a key-based parking reservation facility, which ensures the allocation of the parking lot to the correct person. The system also implements optical character recognition to read license plates and facial recognition for the user, which provides two-way security.

Bagula et al. [36] implemented an SPS using IoT and WSN. The system utilizes an ultrasonic sensor to detect parking lot occupancy and uses RFID tags for vehicle identification and payment facilities. The system implements three kinds of sensor nodes, such as slave nodes or receivers, master nodes or transmitters, and anchor nodes or repeaters. The slave nodes are placed on parking spaces to detect parking lot occupancy. The master nodes gather data of slave nodes and transmit data to the cloud. The repeaters are placed strategically inside the parking facility to increase the coverage area of WSN. The sensor nodes utilize both wired and wireless connectivity.

The author in [37] proposed a WSN based low-cost, and energy-efficient SPS. The SPS is designed to monitor the number of vehicles entering and exiting a parking area rather than detecting vehicle occupancy in every parking lot. The system uses 6LoWPAN over IEEE802.15.4 for communication, which requires less power; thus, the system's energy efficiency increases.

IoT-based SPS for smart cities using RFID and infrared sensors have been developed in [38]. The system uses infrared sensors to detect vacant and occupied parking lots. The sensor data are then sent to the cloud via WiFi. Users can easily access the data through a web application. The SPS uses RFID tags for gate management purposes. Moreover, the SPS provides parking reservations, an online payment facility, and vehicle theft protection.

In [39], an automatic SPS based on IoT has been proposed. The SPS gives real-time parking lot status to the nearest parking areas. The user can access the data via a web application. For data processing and data storage purposes, a centralized server has been used by the SPS.

Orrie et al. [40] presented a WSN based SPS. The system consists of a smartphone application and wireless sensors. The wireless sensor nodes of the system connect via WiFi and Low Power RF transceiver. A user can easily get the location of vacant parking lots by using the smartphone application. The system also provides an RFID-based payment facility.

An automated SPS based on Bluetooth connectivity has been developed. The system uses an automatic mechanical system-based valet parking service that transports vehicles to the unoccupied parking space and retrieves vehicles from the specific parking space without any human intervention. In this system, user authentication to process initiation is done by using Bluetooth connectivity.

The authors in [42] built a prototype for parking space vacancy monitoring based on wireless technologies. The system can be divided into two modules: a master module and a parking lot occupancy checking module. The master module comprises a Laptop Graphical User Interface (GUI), which shows the infrared sensor data. The data is transferred to the laptop via the Zigbee transceiver interface with a PIC microcontroller. The parking space occupancy checking module consists of digital Infrared sensors, which senses if a parking space is occupied or not. The user can see the information on an LCD installed at the entrance of the parking area.

Al-Kharusi et al. [43] presented an SPS based on image processing to detect unoccupied parking spaces. The system uses cameras as sensors to detect parking lot occupancy. The system uses RF communication to send the camera's data to the system's central processing unit. In [40], RF communication with a range of around 1000 feet (300 m) has been achieved using a high gain omnidirectional antenna of 12 dB.

Researchers in [44] implemented a modified WSN and RFID-based SPS. The SPS can provide the real-time parking lot occupancy status for a specific location. The system uses ZigBee communication technology for wireless communication among the wireless sensor nodes. The system has two types of nodes: the monitoring node and the sink node. Each parking lot has a monitoring node that collects data and transmits the obtained data to the gateway sensor node or sink node via ZigBee communication technology. The sink node is connected to the monitoring room via the RS-232 interface.

An SPS based on WSN, IoT, and RFID technology has been developed in [45]. Considering the cybersecurity of the IoT devices, the system utilizes a lightweight cryptographic algorithm. Moreover, the system implements fog or edge computing techniques to manipulate and process the sensitive sensor node data within the network edge. Fog or edge computing techniques reduce computational stress on the central server and decrease the system's energy consumption. The system can provide real-time information on parking space occupancy to the user. Besides, a user can reserve a parking lot and has the option to pay parking fees via online transactions.

In [46], a smart parking system via Optical WSN has been proposed. The system can inform users about the parking lots available in different parking spaces and guide them towards the available parking lots. The system implements a star-based tropology with a polling Medium Access Control (MAC) protocol to communicate with wireless sensor nodes.

The authors in [47] presented a WSN based SPS prototype. The system incorporates WSN nodes, a mobile phone application, a central web server, and an embedded web server. The SPS allows the user to find cost-free parking spaces. Moreover, it provides real-time parking lot status by utilizing wireless sensor nodes. The parking lot data are sent to an embedded web server. From there, the data is again sent to the central web server through which the user can access the data via a smartphone application.

An intelligent image processing based SPS based on has been implemented in [48]. This SPS uses webcams to detect parking lot occupancy. The image data gets processed on an ARM8 based microcontroller. The information is then uploaded to a web server. Users can see the parking lot status via a web application. Moreover, they can see the information on an LCD screen mounted at the entrance gate. Through SMS, any user can book a parking lot. The controller of the system receives the SMS via the GSM module.

A mobile application-based SPS using embedded sensors of a smartphone (such as accelerator, gyroscope) and Bluetooth connectivity has been described in [49]. After initiating vehicle parking at the parking lot, the information disseminates over the target scenario using a combination of internet connectivity to a remote server and Device-to-Device connectivity via WiFi.

Work conducted in [50] proposed an SPS for the urban area, which assigns and reserves parking spaces for users. This SPS provides parking lot suggestions to the user by combining data such as parking charge and vehicle proximity to the destination. The system also includes reservation guarantee, Infrastructure to Vehicle (I2V) communication, Vehicle to Infrastructure (V2I), and parking lot detection.

The researchers in [51] proposed a crowdsourcing-based SPS solution that uses embedded smartphone sensors to detect real-time parking lot availability. The system tracks the driver's trajectory to detect parking lot occupancy. The system applies a waist-mounted Position Dead Reckoning (PDR) method to follow the driver's trajectory. Besides, the SPS implements a map-matching algorithm to calibrate the directional errors in an indoor environment.

Autonomous Vehicle (AV) is a fundamental unit of the smart city and SPS concept. The authors in [52] suggested a scheduling scheme for Long-range Autonomous Vehicle Parking (LAVP). The scheme considers AV's fuel consumption rate and the time it is traveling. The Car Parks (CP) for the AVs are situated outside the city to reduce traffic jams and provide public safety. In this study, the drivers of AVs can select drop-off and pick-up points. These points are to be used by the AVs to go to the nearest CPs and to come back to the pick-up points from the CPs for picking up their drivers.

In [53], an SPS based on Narrow Band IoT (NB-IoT) technology has been proposed, mitigating the excessive amount of power loss due to sensor nodes and the high cost of sensor connectivity. NB-IoT is a new cellular technology that is used for low-power wide-area (LPWA) applications. This SPS integrates a third-party payment platform and provides a smartphone application for the user. Furthermore, it provides a surveillance facility, charge, and information management via a cloud platform.

The authors in [54] devised a prototype for vehicle occupancy detection via Wireless Sensor Network (WSN). The system provides real-time parking lot updates to the user via a smartphone application. Due to the utilization of WSN, the system has ease of sensor placement.

Cynthia et al. proposed an IoT-based SPS integrated with a smartphone application [55]. The system uses infrared (IR) sensors to detect parking lot occupancy. Data obtained from the IR sensors are sent to the cloud platform. Using a smartphone application, the user can access the data. Besides, SPS enables the user to get to the nearest available parking lot location based on the size of the vehicle.

Furthermore, a user can reserve a parking lot by using the system's integrated smartphone application. RFID tags have been utilized to authenticate parking reservations. The parking space owner can monitor the parking lot occupancy status and fix the parking rate.

An IoT-based smart parking application named I_SPARK has been developed in [56]. The application can provide parking space occupancy information for a particular parking area. Users can access the data via web and smartphone applications. The system uses Message Queuing Telemetry Transport (MQTT) protocol for IoT connectivity.

Researchers in [57] suggested a novel smart parking solution for the motorist, which provides information on available parking lots via a smartphone application. The system uses ultrasonic sensors to count the number of vehicles entered and exited from the parking space. A Raspberry pi board processes the sensor's data and sends data to the client-server for storage. Users can access the data from their smartphone applications. Besides, the system provides vehicle guidance to the nearest parking area, where free parking lots are available.

The authors in [58] presented an SPS based on WSN, which provides the parking lot status of a particular parking facility. The system detects parking lot occupancy by using ultrasonic sensors, which are mounted on each parking lot. The data generated by the sensors get processed by Arduino Mega 2560. The processed data get transferred to the sink node of the system. From the sink node, the users can see the parking occupancy status on the monitors placed in different spots of the parking area.

Baroffio et al. proposed IoT and Computer Vision-based SPS [59]. The visual sensor network based on computer vision uses multiple cameras as visual sensors to capture video data of different parking lots, which are processed to detect parking lot occupancy. Due to multiple cameras, an Ad Hoc network is implemented for content acquisition and data transmission to the central controller.

In [60], the authors proposed a Vehicle Ad Hoc Network (VANET) based cloud framework to provide security and privacy-conscious service termed as Parking information as a Service (PIaaS). PIaaS provides SPS details from VANET oriented cloud infrastructure to vehicular nodes in a secure and privacy concerned manner. VANET enabled vehicles along with the Park Side Units (PSUs) communicate parking information known as Parking Mobility Vectors (PMVs) with the Road Side Units (RSUs) via cloud infrastructures. The RSUs are also termed Communication Terminals (CTs). The SPSs implement a geo-location-oriented parking lock encryption, which provides location privacy. The system also includes traffic congestion reports of different routes, vehicle theft protection facilities, and detection of malicious vehicles.

A cloud-oriented SPS based on IoT technologies has been developed in [61]. The SPS provides parking reservation service and uses RFID tags, ensuring only the designated person gets to the parking lot. Moreover, the system uses Number Plate Recognition (NPR) for vehicle security. Also, it can detect oversize and overweight vehicles and restrict them from entering the parking area.

In [62], SPS based on IoT and smart vehicle presence sensors has been proposed. The smart vehicle presence sensor comprises a single-board computer, sensors, LED indicator, beeper, and battery pack. The vehicle presence sensors sense the parking lot occupancy. The monitoring center for the system and the smart vehicle presence sensors and smartphone application monitor, control, and process the data.

The SPS developed in [63] is based on IoT technology. The system aids the driver in finding unoccupied parking spaces in the closest parking area, which reduces the searching effort of the driver. The system additionally provides traffic jam information of different possible routes leading to the SPS facility. The SPS also implements fog computational techniques to decrease the amount of data required for transmission to the cloud for processing and evaluation through a machine learning algorithm.

Researchers in [64] proposed a smart parking solution that integrates a cloud platform based on IoT technology. The system deploys on-site IoT modules that monitor and detect parking lot vacancies. The end-user can get the parking lot status by looking into the smartphone application. The user can also reserve a parking lot using the smartphone application.

In [65], the authors presented an SPS which uses Ultra High Frequency (UHF) RFID and IEEE 802.15.4 technology to detect unoccupied parking spaces in a parking facility. The system aids the driver by providing vehicle guidance to the nearest parking space via a customized smartphone application. Near Field Communication (NFC) based e-wallet facility is provided by the system for paying parking fees. The system also implements the Google Cloud Messaging (GCM) platform to alert the system administrator and the user of the expiration of allocated parking time. Furthermore, the system can inform local law enforcement agencies of any parking lot discrepancies via a custom smartphone application.

A unique SPS based on IoT and Multi-Agent System has been implemented in [66]. It enables parking space providers to rent out their parking spaces. Furthermore, it provides a dynamic pricing scheme for parking space providers. For the drivers, the SPS offers vehicle guidance to parking space and real-time vacant parking lot information.

The authors in [67] presented an SPS which provides pricing, reservation, and dynamic resource allocation. The system ensures a parking guarantee for the user at the lowest possible rate in the shortest amount of time. Moreover, maximum utilization of parking resources is ensured, which provides the highest possible revenue to the parking space owners. The system implements Mixed Integer Linear Programming (MILP) to minimize monetary cost and maximize resource utilization.

Researchers in [68] suggested an IoT-based privacy-preserving SPS platform. The system utilized Elliptic Curve Cryptography (ECC) as an alternative to conventional cryptography based on the public key. ECC is mainly implemented on devices with constrained computational resources (such as speed and memory). Moreover, the system implements Zero-Knowledge Proofs (ZKP) to enhance the system's privacy even more.

An SPS based on Artificial Intelligence (AI) and image processing were designed in [69]. The system deploys ultrasonic sensors for parking lot occupancy detection and uses cameras for number plate recognition which is used for billing and vehicle security. Also, the system provides parking recommendations based on parking fees and parking area distance from the user's location.

An affordable and adaptable smart parking platform using distributed camera networks, advanced deep learning algorithms, and fog computing techniques has been proposed in [70]. The cameras of the system are equipped with motorized heads and zooming lens, which provide vehicle efficient and accurate tracking facility. The system also captures the vehicle number plate image, which calculates precise parking fees for the user and ensures vehicular security.

SPS in [71] is based on distributed intelligence and decentralized decision-providing architecture. Due to this architecture, the system is scalable. The system provides services such as real-time parking lot vacancy detection and vehicle guidance to unoccupied parking spaces.

In [62], the authors deployed a modified CNN for classification of Multiple Input Multiple Output (MIMO) Frequency Modulated Continuous Wave (FMCW) RADAR images to detect vacant parking lots.

The authors in [73] suggested an SPS which utilizes loop detectors as a sensor. The loop detector senses the vehicle on the parking lot. Depending on that data, the system shows parking lot occupancy status on the LCD monitor. The system is suitable for both closed and open parking lots.

A camera and acoustic sensor network-based smart parking surveillance system have been implemented in [74]. The SPS utilized low-cost microphones to detect any acoustic occurrences happening inside of the parking area. When the acoustic sensor network detects any acoustic event, the network quickly localizes the position of the incident and adjusts the cameras to record the situation. The system used a server system, camera system, and acoustic source localization system. The estimated position of the place where the acoustic event occurred can be seen on a map.

## 4 Approaches to smart parking system

Detailed analysis and comparison of the technology methods or approaches used by various SPSs have been provided in this section. Based on the approaches, SPS is divided into 12 different which are covered in sub-sections 4.1, 4.2, 4.3, 4.4, 4.5, 4.6, 4.7, 4.8, 4.9, 4.10, 4.11 and 4.12. Sub-section 4.13 provides an overall summary of the approaches to SPS.

### 4.1 Wireless Sensor Network (WSN) based SPS

WSN can be defined as a network of wirelessly connected sensor nodes that are spatially dispersed and are dedicated to monitoring different environmental aspects such as sound, temperature, pressure, etc. WSN based sensor node comprises various sensors connected to monitor different aspects of the environment. In WSN, all the sensor nodes are connected to a sink node via wireless connection [75, 76]. Nowadays, WSN has received outstanding traction among the SPS developers for flexibility, scalability, and low deployment cost. Due to these benefits, many of the research articles analyzed in this paper utilized WSN as the primary approach towards building SPS.

### 4.2 Multi-agent system (MAS) based SPS

MAS is a self-organizing computer-based system accumulating multiple intelligent agents to solve problems that are pretty difficult for any single system to solve [77, 78, 79]. To develop SPS, various researchers have deployed MAS due to its effectiveness in both closed or indoor and outdoor or open parking lot areas. A significant portion of MAS-based SPS provides computing facilities to the agents, which reduces the data transmission head of the whole system. As a result, the power consumption rate decreases.

### 4.3 Computer vision/image processing based SPS

Computer vision/Image processing based SPS uses different types of camera networks to use image data to extract different information such as parking lot occupancy status [59], license plate recognition (LPR) and face recognition for billing, security issues, and to provide road traffic congestion report [80, 81]. The systems based on computer vision/image processing technologies usually have a high data transmission rate from the camera network to the processing units because these systems are dependent on real-time parking lot video data for feature extraction. These sorts of SPSs are usually suitable for open parking areas because a single camera can capture a significant area in the parking lot. However, these systems are prone to occlusion, shadow effects, distortion, and changing of light.

### 4.4 Vehicular Ad-Hoc network (VANET) based SPS

VANET is based on the Mobile Ad Hoc Network (MANET), where a wireless network of mobile devices is used. SPS utilizing VANET has three main components: Parking Side Unit (PSU), Road Side Unit (RSU), and On-Board Unit (OBU) [82, 83, 84]. The OBUs are installed on the vehicles, PSUs are installed on parking areas, and RSU's are installed beside the roads near the parking areas. This type of system requires a trusted authentication authority that authorizes the vehicle's OBU. If a vehicle is parked inside of a smart parking facility, the OBU of the vehicle provides information to the PSU that the parking lot is booked. Then, this information is transferred to the RSU from the PSU. The vehicles traveling by that road where the RSU is placed can get the information of parking lot occupancy through their OBUs. VANET based smart parking systems are deployable in both closed and open parking lots. But the system is costly and provides erroneous information when a vehicle without an OBU gets inside the smart parking facility.

### 4.5 Internet of Things (IoT) based SPS

IoT is the buzzing technology of the current era, where all devices are interconnected with one another through the internet. Every device interconnected with the internet possesses an unique identifier (UID). These devices can be computational devices, mechanical devices, and digital devices. They can transfer data to without human-to-human or human-to-computer interaction [85, 86, 87]. IoT technology acts as one of the primary key technologies that developers use for SPS. In IoT-based SPS, all the sensors and computational devices are connected through the internet and can transfer data without any human intervention. The internet connection among sensors, computational devices, and storage units can be either through a wired connection or through a wireless connection.

### 4.6 Machine learning (ML) based SPS

ML is a subset of AI that provides a system the ability to learn and improve on a particular task from the datasets or experiences without explicitly programming the system [88]. A machine learning-based SPS analyses the parking lot of data to extract the parking lot status. Moreover, ML and AI-based SPS can predict parking lot occupancy status of the upcoming days, weeks, or even months and provide a dynamic pricing scheme. ML-based systems can monitor traffic congestion of particular roads and offer a smart solution to smart parking spaces [89].

### 4.7 Deep learning (DL) based SPS

DL is a subset of ML and a function of AI which mimics the human brain in terms of data processing and feature extraction to make decisions [90, 91]. DL algorithms detect vacantly occupied and special parking lots in an SPS instead of regular sensors, which reduces the number of sensors and cameras required by the system. DL is also used to predict parking lot occupancy.

### 4.8 Neural Network (NN) based SPS

NN is a combination of algorithms that extracts features and underlying relationships from sets of data through a process that mimics human brain function [30]. In SPS, NN is used for license plate recognition using real-time video data. CNN and machine vision are implemented to detect parking lot occupancy status. CNN's are also capable of providing road traffic conditions of different routes [92].

### 4.9 Fuzzy logic based SPS

Fuzzy logic is a reasoning method that resembles human reasoning. It uses multi-valued logic, which means there is no absolute truth or absolute false value in fuzzy logic [93]. Fuzzy logic is used in SPS for predicting parking lot occupancy status [6]. But the accuracy of the prediction model based on Fuzzy logic would not be that high without validating the prediction result with the real-time data [94]. Therefore, Fuzzy logic, along with machine vision or sensors, improves the accuracy of the overall system.

### 4.10 Global Positioning System (GPS) based SPS

GPS is an essential component of different smart parking approaches. But GPS alone is unable to gather parking lot occupancy status and provide other smart parking facilities. However, GPS can provide a vehicle guidance facility for the user to drive towards vacant parking lots. From GPS data, many systems can forecast parking lot occupancy and road traffic congestion using CNN or DL algorithms [95]. The accuracy of GPS depends on the number of receivers it has. For a single frequency receiver GPS, the accuracy is around 7.8 m. On the other hand, a two-frequency receiver provides around 0.715 m of accuracy. The GPS data is also prone to error if operated inside of a closed parking area. Thus, smart parking systems that use GPS are suitable for open parking lots [50, 51].

### 4.11 Global System for Mobile (GSM) based SPS

GSM is a standard for second-generation (2G) digital cellular networks. GSM standard provides a subsidiary service called SMS. SPS, based on GSM, uses SMS service to reserve parking spots at different parking spaces. Some system also generates unique codes for the users during the reservation process, which are used to authenticate the reservation and ensure that only the designated persons get to park [26].

### 4.12 Bluetooth based SPS

Bluetooth is a wireless communication technology standard that enables data transfer within a short-range. A smart parking system that is wholly based on Bluetooth technology usually has automated valet parking installed. Regular SPS, which does not deploy an automated valet parking facility, requires additional sensors and approaches to get different smart parking facilities [41, 49, 96].

Many smart parking systems use the Crowd-sensing method to gather information about available parking spots in an area. The method uses smartphone sensors (such as Accelerometer, Gyroscope, Magnetometer, and GPS) and applications to gather parking lot information [97].

### 4.13 Classification of reviewed SPSs according to approaches

A summary of the technological approaches used in different research works to develop SPSs and system suitability under different parking lots is given in Table 2. From Table 2, it can be seen that WSN and IoT are the most popular approaches for implementing SPS. Computer vision/image processing can also be considered as a frequently used approach in SPS. The utilization of the remaining technological approaches in SPS is almost the same. SPS can be divided into two major groups based on parking lot suitability: open space and closed parking space. As per parking lot suitability, 17 SPSs are suitable for both open and closed parking lots. While compared individually, 37 SPSs are suitable for indoor/closed parking lots, whereas 26 SPSs are suitable for open space.

**Table 2:** Classification of SPSs according to technological approaches and parking lot suitability.

Ref.	Methods/Approaches												Parking Lot Type	
	WSN	Multi-Agent System	Computer Vision/Image Processing	VANET	IoT	Neural Network	Fuzzy Logic	GPS	GSM	Bluetooth	Machine Learning	Deep Learning	Close Space/Indoor	Open Space/Outdoor
[22]	+				+								+	
[23]		+			+								+	+
[24]	+				+								+	
[25]	+													+
[26]									+				+	+
[27]							+						+	+
[28]			+			+							+	+
[29]			+											+
[30]			+			+							+	+
[31]			+										+	+
[32]	+												+	+
[33]	+				+								+	
[34]					+								+	
[35]					+								+	
[36]	+				+									
[37]	+													+
[38]					+								+	
[39]					+								+	+
[40]	+				+								+	+
[41]										+			+	
[42]	+												+	
[43]			+										+	+
[44]	+												+	
[45]	+				+								+	
[46]	+													
[47]	+												+	+
[48]			+											+
[49]					+					+				
[50]								+					+	+
[51]								+						
[52]													+	+
[53]	+				+								+	
[54]	+												+	
[55]					+								+	
[56]					+								+	
[57]													+	+
[58]	+												+	
[59]	+		+		+									
[60]				+									+	+
[61]			+		+								+	
[62]			+		+									
[63]					+						+		+	+
[64]					+									
[65]	+													
[66]		+			+									
[67]													+	+
[68]					+									+
[69]			+		+						+			
[70]												+	+	+
[71]			+										+	+
[72]						+								+
[73]													+	+
[74]													+	
Total:	18	2	11	1	23	3	1	2	1	2	2	1	37	26

## 5 Implemented sensors in SPS

As per the literature review conducted in section 3, details of sensors used to design, develop, and implement SPSs are expressed in sub-sections 5.1, 5.2, 5.3, 5.4, 5.5, 5.6, 5.7, 5.8, 5.9, 5.10, 5.11 and 5.12. Sub-section 5.13 provides an overall summary of the sensors used in SPSs.

### 5.1 Infrared (IR) sensor

IR sensor is an electrical device that detects and measures IR radiation emitted from an object. Any object that has a temperature higher than 5° or above emits IR radiation. IR sensors are mainly used for motion detection and temperature measurement purposes. IR sensors can be categorized into two types: Active Infrared Sensor and Passive Infrared Sensor.

#### 5.1.1 Active IR sensor

Active IR (AIR) Sensor emits infrared radiation and detects the radiation reflected from any object that stays in its proximity. This type of IR sensor has two components: Light Emitting Diode (LED) and receiver. LED emits radiation and receiver detects reflected IR radiation. An active IR sensor is sensitive to rain and snow. Thus, it is more suitable for object detection in indoor environments such as closed or indoor car parking facilities. High deployment and maintenance cost is a significant issue associated with Active IR Sensor [98, 99].

#### 5.1.2 Passive IR (PIR) sensor

Passive Infrared (PIR) Sensor does not emit IR radiation. Instead, it detects changes in radiation from its surroundings. PIR sensors are mainly used for detecting objects. In SPS, it is primarily used for parking lot occupancy detection. However, like the AIR sensor, the PIR sensor is sensitive to environmental changes such as rain and snow. Thus, it is not suitable for open parking lots. Moreover, PIR sensors are also expensive to deploy and maintain [98, 99].

### 5.2 Cellular Sensor

Cellular sensors are the sensors that are installed inside a smartphone. Although a smartphone might comprise many sensors, SPS, Accelerometer, Gyroscope, and Magnetometer are the mainly used sensors. These sensors are used for detecting the user's motion, orientation, and direction [49, 51].

### 5.3 Magneto-Resistive (MR) sensor

MR sensors are designed to detect the applied magnetic field without using any electrical contact. The principle of the MR sensor is very straightforward. When a magnetic field is applied, a change of resistance in any electrical conductor occurs that permeates. Changes in resistance depend on the orientation of the magnetic field lines. MR is mainly used for vehicle detection in parking lots [22].

### 5.4 Acoustic array sensor

An acoustic array sensor detects sound or vibration of specific frequencies to determine the distance and direction of the sound source that created the sound or the reflector that reflected the sound. This type of localization technique is known as the passive acoustic location technique [74, 100]. In SPS, Acoustic Array Sensor is used to detect parking lot vacancy and for surveillance purposes.

### 5.5 Ultrasonic sensor

Ultrasonic sensor uses acoustic waves in the range of 25 kHz to 50kHz to detect any nearby object that reflects the acoustic wave [100]. This sensor is best suited for indoor applications due to not having the susceptibility to work under environmental changes such as snow and rain. Therefore, ultrasonic sensors are used for closed and indoor parking facilities where these sensors are usually mounted on the ceiling. Ultrasonic sensors can detect vehicles. Moreover, with proper implementation, this sort of sensor can segregate between a vehicle and a passer-by. Ultrasonic sensors are cheap and have a low maintenance cost.

### 5.6 Camera

Using a camera or a network of cameras for vehicle detection and parking lot surveillance is widely adopted by many SPS researchers. Many researchers have used cameras and different computational tools (such as computer vision, image processing, etc., techniques) to detect license plates of vehicles for billing, reservation, and authentication. SPS using a camera or network of cameras provides a robust parking solution for users. However, camera-based SPS often tends to be expensive for both deployment and maintenance [28, 29, 30, 31].

### 5.7 Inductive Loop detector

An inductive loop detector (also known as an Inductive loop traffic detector or Vehicle loop detector) is a vehicle detection method that utilizes the electromagnetic induction principle. This type of detector is installed under the road to detect vehicles above it. These detectors, along with some computational techniques, can classify different kinds of vehicles [73, 101]. Vehicle loop detectors are expensive and have a high installation cost. This sort of sensor is suitable for both open and closed parking lots.

### 5.8 Light Detection and Ranging (LIDAR)

LIDAR is a technique that uses LASER light to calculate the distance by illuminating the object of interest and measuring the reflected light with the sensor. A 3D representation of the object of interest can be made by measuring the time it takes to get the reflected light and light's wavelength [70]. For SPS, LIDAR is mainly used for vehicle detection.

### 5.9 Microwave Radio Detection and Ranging (RADAR)

Microwave RADAR uses electromagnetic waves in the microwave spectrum to determine the target object's velocity, distance, and angle. But Microwave RADAR can only detect moving objects [102]. To determine both stationary and moving objects, Doppler Microwave RADAR can be used [96]. With 2D images generated by radar sensors and AI, an SPS can substitute a video camera. Moreover, by utilizing the data obtained from Microwave RADAR, a CNN can be trained to predict parking lot occupancy status. Environmental changes do not affect this type of sensor. Thus, they are suitable for both open and closed parking lots. However, this sort of sensing technique is expensive to deploy and maintain.

### 5.10 Magnetometer

A magnetometer can sense the existence of any vehicle by sensing the change of electromagnetic fields around it. Magnetometers are installed beneath each parking lot to sense the presence of any vehicle [71]. Magnetometers are insensitive to environmental changes, making them suitable for both closed space and open space SPS.

### 5.11 Agent

An agent is a part of a multi-agent system that encompasses sensors, processors, and other tools. An agent itself can be considered a small system part of a more comprehensive network of systems that can generate, process, and transmit data to capture a bigger picture of the environment [23, 66].

### 5.12 Radio Frequency Identification (RFID) sensor

RFID technology uses electromagnetic fields to identify and track an object. RFID technology uses a radio transponder that encompasses an RFID receiver and an RFID tag. When the RFID tag gets scanned by the RFID receiver, it transmits digital data stored inside it. The receiver receives the data for object identification. RFID technology is very commonly used in smart parking systems for vehicle and user identification [24, 32, 103, 104].

### 5.13 Summary of utilized sensors

Table 3 summarizes the utilization of different sensor technologies by other SPS papers. A careful study of Table 3 shows the widespread utilization of IR Sensor, Acoustic Array Sensor, Camera, and Inductive Loop Detector in SPSs. Low implementation and maintenance cost is the main reason behind the popularity of IR Sensor, Acoustic Array Sensor, and Inductive Loop Detector in SPSs. The installation of a Camera might seem to be an expensive move towards SPS implementation. However, the Camera coverage area is relatively high compared to the other sensors, for which it becomes possible to cover a large. area with few Cameras Moreover, the Camera provides live surveillance of parking space, eliminating the need to install several other security protocols in a system. Due to the multitudinous functionalities, the Camera remains one of the frequently used sensor topologies in SPS.

**Table 3:** Sensor topologies utilized in various SPSs.

Reference no.	Infrared (IR) Sensor	Cellular Sensor	Magneto-Resistive (MR) Sensor	Acoustic Array Sensor	Ultrasonic Sensor	Camera	Inductive Loop Detector	LIDAR	Microwave RADAR	Magneto Meter	Agent	RFID Sensor
[22]			◘									
[23]												◘
[24]				◘		◘						
[25]				◘								
[26]												
[27]												
[28]							◘					
[29]							◘					
[30]							◘					
[31]						◘	◘					
[32]				◘		◘						
[33]	◘											
[34]						◘						
[35]							◘					
[36]				◘		◘						
[37]	◘											
[38]	◘			◘								
[39]							◘					
[40]				◘								
[41]												
[42]	◘											
[43]							◘					
[44]				◘		◘						
[45]				◘		◘						
[46]	◘						◘					
[47]	◘											
[48]							◘					
[49]		◘										
[50]				◘		◘						
[51]		◘										
[52]												
[53]			◘									
[54]						◘						
[55]	◘			◘								
[56]	◘											
[57]						◘						
[58]						◘						
[59]							◘					
[60]												
[61]	◘			◘			◘					
[62]						◘	◘					
[63]	◘											
[64]	◘					◘						
[65]				◘								
[66]												◘
[67]												
[68]			◘	◘								
[69]						◘	◘					
[70]							◘		◘			
[71]				◘			◘				◘	
[72]							◘			◘		
[73]								◘				
[74]					◘		◘					
Total	11	2	3	14	1	14	17	1	1	1	1	2

## 6 Networking technologies

One of the critical parts of smart parking systems is networking. Networking brings the data generated by the sensors to the processing units or brings the processing unit's processed information to the end-users. In other words, it creates bridges among the sensors, processors, and end-users. In SPS, the network can be divided into two parts: Sensor Network and User Network. Networking and communication technologies used by various SPS are summarized in Table 4. Specification of the communication protocols has been provided to have an overall idea of the SPS network and communication technologies.

**Table 4:** Deployment of various communication/networking technologies and utilization of different user interfaces in SPS.

Ref. no.	Communication/Networking Technologies in SPS		User Interface in SPS		
	Sensor Network	User Network	Web Application	Smart Phone Application	Vehicle Information Communication System (VICS)
[22]	ZigBee	WLAN (802.11a/b/g)	✓		
[23]	Contract Net				✓
[24]	ZigBee	WiFi/3G Networks		✓	
[25]	WiFi/Ethernet		✓	✓	
[26]		GSM	✓		
[27]				✓	
[28]	Wired		✓		
[29]	Wired				
[30]	Wired		✓		
[31]	Wired				✓
[32]	ZigBee				
[33]	WiFi (ESP8086)			✓	
[34]	WiFi (ESP8266)			✓	
[35]	WiFi			✓	
[36]	IEEE 802.15.4/ZigBee				
[37]	6LoWPAN (over IEEE 802.15.4)			✓	
[38]	WiFi		✓		
[39]	WiFi		✓	✓	
[40]	WiFi & CC1101 Low Power 1GHz RF Transceiver			✓	
[41]		Bluetooth		✓	
[42]	ZigBee		✓		
[43]	RF Communication				
[44]	ZigBee				
[45]	RF Communication			✓	
[46]	RF Communication (MAC Protocol)				
[47]	WiFi + ZigBee	WiFi/3G Network	✓	✓	
[48]	LAN (Ethernet)	GSM	✓		
[49]		D2D via WiFi Direct/3G/4G Networks		✓	
[50]	LAN (Ethernet) + ZigBee	Cellular Networks		✓	✓
[51]		Cellular Networks		✓	
[52]	Cellular Networks	Cellular Networks			
[53]	Cellular Networks	Cellular Networks	✓	✓	
[54]	ZigBee		✓	✓	
[55]	WiFi			✓	
[56]	Wired/Wireless	Cellular Networks	✓	✓	
[57]	Wired	Cellular Networks		✓	
[58]	ZigBee				
[59]	IEEE 802.15.4				
[60]	DSRC (Dedicated Short Range Communication)/Wave 802.11p	3G/4G Networks			✓
[61]	WiFi (ESP8266)	WiFi/3G/4G Networks		✓	
[62]	WiFi			✓	
[63]	Wireless (Bluetooth)	3G/4G network		✓	
[64]	WiFi (ESP8266)			✓	
[65]	Zigbee			✓	
[66]	IEEE 802.15.4		✓		
[67]	Wireless/Wired			✓	
[68]	GPRS & IEEE 802.15.4 With Digimesh	WiFi/GPRS		✓	
[69]	WiFi & 3G/4G/EDGE/GPRS			✓	
[70]	WiFi mesh		✓		
[71]	CoAP, ETSI ITS G5, 6LoWPAN (over IEEE 802.15.4)		✓	✓	
[72]	WiFi				
[73]	RF Communication				
[74]	IEEE 802.15.4				
Total			16	29	4

### 6.1 Sensor network

Sensor Network is the part that connects the sensors to the processing unit for data transmission. The sensor network can be wired or wireless. For wireless communication, SPS can use many standard wireless technologies such as ZigBee, WiFi, Local Area Network (LAN), Direct Short Range Communication (DSRC), Constrained Application Protocol (CoAP), 3G/4G cellular networks, Enhanced Data for GSM Evolution (EDGE), General Packet Radio Services (GPRS), IPv6 over Low Power Wireless Personal Area Networks (6LoWPAN), etc.

### 6.2 User network

The user network connects the end-users to the SPS for data visualization, parking reservation, billing, and other related works. User Network is mainly based on wireless connectivity such as 3G/4G cellular networks, WiFi, Device to Device (D2D) via WiFi, GPRS, GSM, Wide Local Area Network (WLAN), etc.

## 7 User interfaces

To communicate with SPS, users may use different interfaces (such as web applications, smartphone applications, and Vehicle Information and Communication System). A description of various user interfaces used in SPSs is presented in the following sub-sections.

### 7.1 Web application based SPS

To provide remote access to the end-users, many SPSs use web applications based on Hyper Text Transfer Protocol (HTTP) and Transmission Protocol (TCP)/Internet Protocol (IP) protocols. Web applications of SPS usually provide a graphical user interface (GUI) to the end-user. The web application offers services such as real-time parking lot status of the parking area, guidance to the nearest parking lot or parking area, parking spot reservation facility, online payment facility, etc.

### 7.2 Smart phone application based SPS

A significant percentage of SPS deploys Android or IOS applications for the end-users to provide them information regarding the smart parking facilities. Like web-based applications, smartphone applications also provide a GUI for the user to interact with the system. Also, to receive information on real-time parking lot status, get vehicle guidance to the nearest parking area or a lot, reserve parking lot, retrieve parking lot information, and pay parking fees via online services or smartphones' Near Field Communication (NFC) technology.

### 7.3 Vehicle information and communication (VICS) based SPS

VICS is a technology used for delivering traffic and travel information to the driver via a monitor mounted on the vehicle's dashboard. The VICS uses wireless technologies such as Frequency Modulation (FM), microwaves in the Industrial Scientific and Medical (ISM) band, Data Radio Channel (DRC), IR, and Radio Data System (RDS) to transmit and receive data. In SPS, VICSs are used to get information on the nearest parking area and traffic congestion. In some cases, they can also be used for parking reservations.

### 7.4 Synopsis of applied communication technologies and user interfaces in SPS

Table 4 illustrates the types of communication/networking technologies used by various SPSs and provides information on the different user interfaces provided by SPSs. It is evident from Table 4 that smartphone application-based SPSs are the most common sort of SPS, whereas VICS-based SPS are the least utilized.

## 8 Computational approaches in SPS

The computational unit is a vital part of an SPS. The computational unit can be a physical unit placed somewhere inside the parking area or can be done over a cloud platform. The reviewed SPSs implemented the following computational methods.

### 8.1 Big Data

Large-scale SPS generates a massive amount of data, usually for which Big Data is used as their computational method. Big Data usually refers to a colossal amount of structured and unstructured data, which are way too big and complex for traditional data processing application software to deal with. It provides a way to handle the colossal amount of data and generate insights. Large SPS can implement Big Data, either on-premise or on cloud service [105, 106, 107, 108].

### 8.2 Cloud computing

SPS, which requires out-of-the-site sensor data processing and storage units, usually relies on cloud computing. Cloud computing provides on-demand cloud storage and data processing capability to the system without any direct monitoring by the user [109]. Usually, the system based on IoT technology tends to use cloud computing [22, 38, 39].

### 8.3 Fog computing

Fog computing refers to an architecture that optimizes the edge device's capabilities to process, store, and communicate with the end router over internet technology. This architecture can be perceived in both cloud computing and Big Data. SPS using a decentralized approach tends towards data process and storage using Fog computing [110, 111, 112]. Using Fog computing, SPS can reduce the amount of data required to be transmitted, making the systems more energy efficient.

## 9 SPS classification based on services

In current times, there are many SPS available throughout the globe. Based on the systems' services, the SPSs can be categorized into five major groups which are covered in sub-sections 9.1, 9.2, 9.3, 9.4 and 9.5. Sub-section 9.6 presents a synopsis of the provided services in SPS.

### 9.1 Parking guidance and information system (PGIS)

PGIS provides real-time parking lot information on controlled parking areas. It is an important part of the Intelligent Transportation System (ITS) and a supplement of the vehicle guidance system [113]. In PGIS, sensors are placed at the entrances and exits to detect and count the number of vehicles entering or exiting the parking area. By detecting the number of vehicles entering and exiting available parking, number of remaining parking lots is calculated. Different computational technologies (ML, CNN, etc.) are used to determine the parking lot status along with the parking lot sensors. Users can see the processed information via different interfaces. Due to the information, users can have choices in selecting suitable parking lots. PGIS system also provides vehicle guidance to the users, which leads them towards the designated parking space.

### 9.2 Transit based information system

Transit-based smart SPS provides parking lot information near the public transportation facilities. Moreover, it presents public transportation schedules to motivate users to use public transportation services, which reduces traffic congestions, environmental pollution, and fuel consumption [114]. Transit Based Information System is implemented using vehicle detection sensors and other computational techniques to obtain information on parking lot occupancy in real-time. Furthermore, it provides vehicle guidance to the chosen parking lot via variable message signs along the highway.

### 9.3 Smart payment system focused parking

This kind of smart parking system deploys advance payment facilities for the users instead of conventional parking meters. Due to the deployment of an advanced payment facility, the system paves the way to fast and convenient payment collection. As a result, the time required for collecting parking fees and fines reduces significantly. Moreover, quick access to upcoming users is ensured in this system. Therefore, parking lots are unoccupied for a less amount of time. The system may use contactless methods like NFC, RFID, and smart cards [115]. The system may also deploy secure online payment methods for parking fee collection via smartphones or web applications. For calculating the parking fees, the system uses the same sensors, which detect the parking lot occupancy.

### 9.4 E-parking system

E-Parking system utilizes advanced technological features to bring different smart parking facilities under one platform [116]. E-Parking systems are based on smartphone or web applications. Anyone having the app downloaded to their smartphone may use the facilities remotely. At first, for using the smartphone application, a user needs to sign up using the required personal and vehicle information. Once after completing registration, a user can access the E-Parking facilities. E-Parking system provides various facilities to the user, such as providing information about the occupancy status of different parking lots situated in other parking stands, vehicle guidance to the nearest parking stand, reservation facility, various payment methods, vehicular security, and also lot retrieval facility.

### 9.5 Automated parking system (APS)

APS is an automated mechanical system that automatically parks vehicles without any human intervention [59]. This system requires Automated Parking Facilities (APF), where the user checks in with the vehicle and places it in the allocated bay. From there, a mechanical system automatically parks the vehicles in a specific parking spot. To retrieve the vehicle, the user must sign in to the system and pay the parking fees. After the payment, the system brings out the vehicle from its parking spot. APS provides the user the information on the number of available parking lots inside the parking space via digital display monitors. Moreover, APS classifies vehicles and places them in different specified parking lots. Due to no human intervention in the parking process, the vehicles are less prone to damage due to parking. APS provides efficient use of parking spaces.

### 9.6 Synopsis of services provided by different types of SPSs

The services provided by various SPSs are summarized in Table 5. Table 5, Parking Reservation, is the service where the user can reserve a parking lot in the designated area in advance by using the GUI provided by SPS. Vehicle Guidance aims to guide the user towards the allocated/free parking lot in a parking area. Online Payment is the SPS service through which the user can pay the parking fees online. Gate Management service is the management of various gates in a parking area. It provides the authority of the facility to open or close the desired gates in the parking area to control the traffic flow. Parking Supervision means parking management from both user and authority points of view. Lot retrieval means providing specific guidelines to the user to find the parked vehicle.

**Table 5:** Smart Parking Systems and the service they provide.

Reference no.	Parking Reservation	Security	Vehicle Guidance	Online Payment	Gate Management	Parking Supervision	Lot Retrieval
[22]	∗	∗			∗	∗	∗
[23]	∗	∗	∗	∗			∗
[24]	∗	∗	∗		∗	∗	∗
[25]	∗	∗	∗	∗		∗	
[26]	∗	∗			∗		∗
[27]			∗			∗	
[28]		∗				∗	
[29]		∗				∗	∗
[30]		∗				∗	
[31]			∗			∗	
[32]	∗					∗	∗
[33]						∗	
[34]	∗			∗		∗	
[35]	∗	∗		∗			
[36]	∗	∗				∗	∗
[37]						∗	
[38]	∗	∗		∗	∗	∗	∗
[39]			∗			∗	
[40]			∗	∗		∗	∗
[41]		∗		∗		∗	∗
[42]						∗	
[43]		∗				∗	
[44]		∗	∗			∗	∗
[45]	∗	∗				∗	
[46]			∗		∗	∗	
[47]						∗	∗
[48]	∗	∗	∗	∗		∗	∗
[49]							
[50]	∗		∗	∗	∗	∗	
[51]				∗			
[52]						∗	
[53]	∗			∗		∗	
[54]	∗					∗	
[55]	∗		∗			∗	
[56]						∗	
[57]			∗			∗	
[58]						∗	
[59]						∗	
[60]		∗	∗			∗	
[61]	∗	∗		∗		∗	
[62]	∗		∗			∗	
[63]			∗				
[64]	∗			∗		∗	
[65]	∗	∗	∗	∗		∗	
[66]	∗		∗	∗		∗	
[67]	∗		∗			∗	
[68]		∗	∗			∗	
[69]		∗	∗			∗	
[70]		∗	∗			∗	
[71]			∗			∗	
[72]						∗	
[73]						∗	
[74]		∗					
Total	22	23	23	15	6	46	13

## 10 Discussion

Based on the comprehensive analysis of SPS presented in sections 3, 4, 5, 6, 7, 8 and 9, discussions are presented in the following sub-sections.

### 10.1 Discussion on SPS approaches

**Figure 5:**
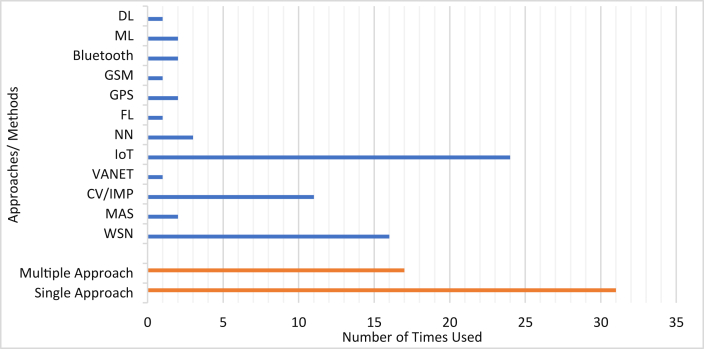
Technological approaches of different smart parking systems.

After analyzing the contents presented in Table 2, a graphical representation of SPS approaches versus the usage of approaches has been illustrated in Figure 5. With careful observation of Figure 5, it can be noticed that around 17 SPSs used multiple approaches rather than sticking to just one. Moreover, it can be seen that 31 research articles followed only a single approach to develop their SPSs. As per the analysis of Figure 5 it can be easily be referred that IoT-based SPSs are the highest ones to be deployed due to their lower deployment and maintenance cost. WSN based SPS approach remains in second place. WSN system is wirelessly connected, for which it requires less amount of wiring. As a result, the overall expenditure for the deployment of the system can be minimized. Although a Computer vision (CV) or image processing (IMP) system requires a smaller number of sensors for vehicle detection, the system requires massive installation costs associated with camera network. However, this kind of camera-based system requires less maintenance. As per review illustrated in Figure 5, ML, DL, GSM, and VANET based systems have the lowest usage. High installation cost and requirement of OBU installation in vehicles are the primary reason for the low usage factor of VANET based SPS.

**Figure 6:**
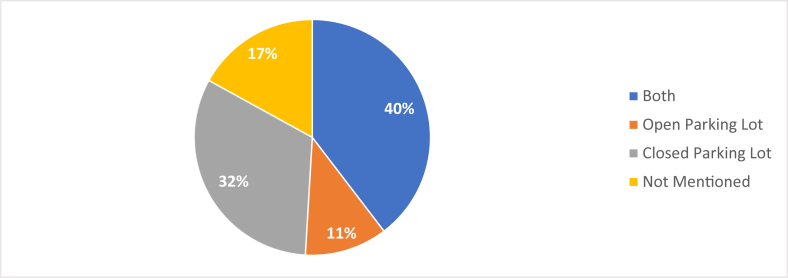
Suitability of smart parking systems.

From Table 2, a pie chart can be generated, as presented in Figure 6. Figure 6 provides statistical information on the suitability of different parking lots. From the pie chart, it can easily be inferred that 40% of the research articles have developed SPS suitable for both open and closed parking lots. From Figure 6, it can also be seen that 32% of the SPSs are ideal for only closed and indoor parking lots, and only 11% of the total SPSs are suitable for open parking lots. From the chart, it can be seen that 17% of the paper did not discuss their SPSs parking lot suitability.

### 10.2 Discussion on sensors used in SPSs

**Figure 7:**
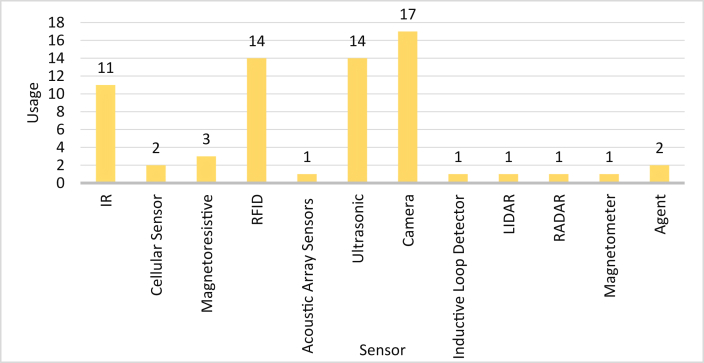
Usage of different sensors in smart parking systems.

From Table 3, the usage data of different sensors by various SPSs are represented in Figure 7. From the figure, it can be seen that the Camera is the highest used sensor. Although the camera network has a high deployment cost, it can provide a wide coverage area, reducing the number of sensors required in a given area. Many of the systems that used cameras deployed different computational techniques (Such as ML, DL, etc.) to detect parking lot occupancy. As a result, the necessity of vehicle detection sensors (such as IR sensors, ultrasonic sensors, etc.) becomes low to almost zero. Camera networks also provide surveillance facilities and license plate detection, which improves overall security. On the other hand, ultrasonic and RFID sensors are the second most used sensors. Ultrasonic sensors are mainly used for vehicle detection to provide real-time parking lot occupancy status. But this type of sensor is prone to environmental changes. That is why this type of sensor is more suitable for closed parking facilities. RFID sensor is another frequently used sensor. Although the RFID sensor cannot provide real-time vehicle occupancy status, it can make payments and provide vehicle security and authentication. Here, many of the other sensors were used a smaller number of times due to less cost-effectiveness or higher installation and maintenance cost. From the graph, it can also be noticed that IR sensors come third in usage. IR sensors are cheap, but their readings are sensitive to environmental changes. As a result, they are suitable for closed or indoor parking facilities.

### 10.3 Discussion on communication protocols and user interfaces in SPS

Considering the data provided in Table 4, it is evident that almost all of the reviewed papers chose wireless connectivity over wired connectivity for network and communication purposes in SPSs. Deployment is much easier and cheaper in wireless communication protocols compared to wired connection. Moreover, wireless communication based network can be easily expanded with the expansion of SPS coverage area.

From Table 4, it can be noticed that almost all of the SPSs had a user interface for the users to communicate with the systems from a distance in order to receive information such as parking lot status, fastest route to the parking lot, etc. Moreover, it is observed that usage of smart phone application based SPS is the highest. Although web application based SPS can be accessed through smart phones, the user needs to remember or copy the web address in order to gain acces to the system. In modern world, the complexities in everyday life have greatly increased due to which remembering website of SPS can be considered as a hassle to the user. On the other hand, smart phone application has specific name and icon in the main screen of smart phone due to which it becomes more easier to find and use the SPS application. For this reason, smart phone application based SPS is getting popular.

### 10.4 Discussion on various services provided in SPSs

Based on the information presented in Table 5, it can be seen high priority has been given to Parking Supervision. A moderate level of emphasis has been provided to Parking Reservation, Security, and Vehicle Guidance. Gate Management receives the least priority in SPSs. Although the number of SPS providing online payment facilities is not relatively high, the necessity for this facility is likely to be increased in the upcoming days due to recent progresses in online transactions.

### 10.5 Future of SPS

After careful observation of the tables and illustration, it can be seen that much of the SPSs developers opted for a single approach rather than multiple approaches. Recent literature, which used multiple approaches to develop their SPSs, tends to provide more services than a single approach based on SPS. As nowadays, users are more concerned about the services they can get for the money they spent; a large number of the future SPSs will be using a combination of different approaches instead of one. Moreover, with the rapid progression of internet and communication technology, there is a reduction in the cost of sensors, networking tools, and computational devices, which have led to a decrease in the complexity of developing multi-approached SPSs. Due to the reduced prices of sensors, networking tools, and computational devices, IoT has gained much traction among SPS developers. Although IoT itself can be an approach towards developing SPS, in this study, it can be seen that most of the multi-approached SPSs used IoT as a connector. With the advent of the smart city concept, the development of the IoT network has increased tremendously. SPS being part of the smart city concept, connecting the smart parking facilities with the existing IoT network is much more viable for developers. Therefore, it can be reckoned that, in the future, most of the SPSs will be based on multiple approaches where IoT will play the key role.

## 11 Conclusion

Due to the rapid increase in urban population and unplanned urbanization, there is a decrement in the number of urban parking spaces and an increment in traffic congestion. As a result, smart parking becomes the subject of interest for both researchers and urban planners. This paper provides a bird's eye view of smart parking systems deployed by different researchers. The paper systematically talks about different approaches taken by the researchers to develop their smart parking system and their suitability for different parking lots. The approaches for SPS have been classified into 12 major groups, and an extensive comparison among the approaches has been provided to determine their strengths and weaknesses. The paper also provides information about different smart parking sensors and their usage for different conditions. A data table giving information on various sensors has been provided to determine the constantly used sensors used in SPS systems. Moreover, classification of SPS as per user interfaces, networking technologies, computational approaches, and services have been provided. These classifications provide a clear insight into SPSs from different angles and aspects. Besides, the study provides an elaborate discussion on the advantages and shortcomings of different types of SPSs in addressing various kinds of problems thrown at the systems.

As per comprehensive review and analysis conducted in this research, it can be observed that multi-approach based SPS will dominate in future smart cities where the IoT will work as the backbone. The user interface will be smart phone application based where services such as parking supervision, online payment, parking reservation and vehicle guidance will be common features of SPS. The selection of sensors in SPS will vary depending on several indoor and outdoor conditions. However, ease in installation, privacy, sensing method and sensor coverage area will be the major issues of concern in case of sensor selection. Security in data communication protocols will also remain a major source of concern in future SPS systems. As a result, more emphasize will be provided in wireless communication protocols to ensure data security.

## Declarations

### Author contribution statement

All authors listed have significantly contributed to the development and the writing of this article.

### Funding statement

This research did not receive any specific grant from funding agencies in the public, commercial, or not-for-profit sectors.

### Data availability statement

No data was used for the research described in the article.

### Declaration of interests statement

The authors declare no conflict of interest.

### Additional information

No additional information is available for this paper.
